# National profile of foot orthotic provision in the United Kingdom, part 1: practitioners and scope of practice

**DOI:** 10.1186/s13047-017-0215-4

**Published:** 2017-08-01

**Authors:** CJ Nester, A Graham, A Martinez-Santos, AE Williams, J McAdam, V Newton

**Affiliations:** School of Health Sciences, Brian Blatchford Building, University of Salford, Salford, UK

## Abstract

**Background:**

Foot orthoses have been advocated in the management of a wide range of clinical foot and lower limb problems and are within the scope of podiatry, orthotic and physiotherapy practice. Previous reports into the provision of orthoses have consistently identified significant issues with services and devices, but data were never specific to foot orthoses. The aim of this first of a series of papers was to report the first ever national multi professional profile of foot orthosis provision in the United Kingdom.

**Methods:**

Quantitative and qualitative data were collected from podiatrists, orthotists and physiotherapists via an online questionnaire. The topics, questions and answers were developed through a series of pilot phases. The professions were targeted through electronic and printed materials. Data were captured over a 10 month period in 2016.

**Results:**

A total of 499 responses were included in analysis, including 357 podiatrists, 93 orthotists and 49 physiotherapists. The results reveal wide ranging practices across podiatrists, orthotists and physiotherapists, provision of orthoses through different health care departments (uni and multidisciplinary), for different health conditions (acute and chronic), and involving different types of orthoses (prefabricated and customised).

**Conclusion:**

Foot orthoses in the United Kingdom are provided in areas of well recognised health and rehabilitation priorities. A wide range of orthotic devices and practices are employed and different professions provide foot orthoses in different ways.

**Electronic supplementary material:**

The online version of this article (doi:10.1186/s13047-017-0215-4) contains supplementary material, which is available to authorized users.

## Background

Foot orthoses modify how load is applied to the plantar surface of feet and thereafter affect the stresses experienced by foot tissues. They are advocated for management of a wide range of foot problems including pain in the heel, Achilles tendon, midfoot joints and metatarsal area [1–5]. Since the foot is coupled to the rest of the lower limb, foot orthoses have also been considered for knee, hip and back problems [6–10]. Foot orthoses are advocated in various national and international practice guidelines, including those for management of feet affected by diabetes [11], rheumatoid arthritis [12, 13], and knee arthritis [14]. As such, the use of orthoses is within the scope of practice for a wide range of practitioners, including podiatrists, orthotists and physiotherapists [15, 16].

Reports into the provision of orthoses in the United Kingdom have consistently identified significant issues with the cost and quality of the devices, cost of and access to services, and models of service delivery [17–20]. However, these reports typically consider all types of orthoses and identifying specific issues with foot orthoses is difficult. This means that there are unanswered questions about where and how foot orthoses are provided, to whom and by whom, for what purpose, at what cost and towards what outcomes. This is complicated by the fact that different professions are involved, and they work in different and overlapping contexts (e.g. different locations, departments, and contractual arrangements), across many patient groups (e.g. diabetes, musculoskeletal, children, etc.) and with different levels of autonomy (e.g. single practitioners vs. multidisciplinary teams) and budgets (e.g. fixed tariffs vs. open market).

The lack of data describing foot orthosis services has recently received national attention [18] and may mean that policy, practice and service innovations are poorly informed. For example, the nature and scope of services to be commissioned might be difficult to describe, variations in practice between clinicians or organisations cannot be put into a national context, and the factors affecting variations in practice (between individuals or organisations) are not understood and thus cannot be managed. The literature that does exist on foot orthosis practice has focused on specific clinical conditions, foot type and prescription details, but not services or practitioners providing the orthosis, and has been specific to podiatrists [16, 21].

The aim of this work was to provide the first ever national multi professional profile of foot orthosis provision in the United Kingdom. Specifically, to determine who are providing foot orthoses, their working context, which patient groups receive orthoses, what orthoses are provided and why, at what cost, and what factors affect practice. In this first of a series of articles we report the national picture concerning providers of orthoses, the patients they treat, the orthoses provided, and factors affecting practice.

## Methods

The approach was to collect quantitative and qualitative data via a questionnaire suitable for online completion and that would capture the orthosis practice of podiatrists, orthotists and physiotherapists in the United Kingdom. The survey was approved by the institutional ethics committee (HSCR14/125.) and online data capture provided means of securing informed consent.

### Survey development and piloting

The development of the draft survey questionnaire was led by four academic staff with professional backgrounds in orthotics and podiatry practice and services, and foot health research. They invited seven clinical podiatry and orthotist practitioners to form a steering group. These professionals worked through several iterations to identify topics for the survey, sample questions and responses, and identify appropriate terminology. Once this process had reached a general consensus the outcome was taken to be a draft survey questionnaire fit for pilot testing.

The draft survey questionnaire was implemented within the Bristol Online Survey platform (https://www.onlinesurveys.ac.uk/) and piloted with an invited group of practitioners comprising podiatrists, orthotists and physiotherapists. A total of 16 practitioners were invited of which 6 (all podiatrists) completed the pilot. Following amendments made in response to feedback, a further pilot test was conducted with 13 further practitioners; 9 podiatrists, 3 orthotists and 1 physiotherapist. The questions were then rationalised down from 90 to 60 based on feedback from the pilot phases.

The final survey questionnaire consisted of five main sections with 60 questions in total (Additional file 1: Appendix A).

Part 1: “about you” – including participant demographics, time since qualifying, professional role, and qualifications.

Part 2: “your practice” – public/private sector nature of where the participant works, and the department and facilities available.

Part 3: “your patients” – patient groups the participant provides foot orthoses to and outcomes that the participant and patient are aiming to achieve.

Part 4: consisted of three sub-sections related to “your practice”.

Part 4.1: “your practice – general orthotic prescribing habits”.

Part 4.2: “your practice – the use of pre-fabricated orthoses” (optional section).

Part 4.3: “your practice – the use of bespoke/custom-made orthoses” (optional section).

Part 5: “other information” - additional information about current prescribing habits, knowledge of orthosis practice, and views about what should/will change in respect of orthotic devices and practice.

### Sampling

The sampling strategy sought to create widespread awareness of the survey amongst the target professions. Notifications were sent between January and June 2016. All correspondence was standardised in terms of language and provided a hyperlink to the online survey. Press releases were provided for inclusion in the national journal/magazines of the Society of Chiropodists and Podiatrists, Chartered Society of Physiotherapists, and the British Association of Prosthetists and Orthotists. Specialist interest groups were also targeted, including MSK UK (Facebook group), National Health Service orthotic managers groups, and related informal clinical effectiveness/speciality groups. A total of 20 commercial suppliers of orthoses related materials and products (to podiatrists, orthotists and physiotherapists) were contacted. These all operated nationally and were companies that would generally be known to the professionals being targeted. They agreed to send out electronic or printed notifications to their customers. Notices were also distributed at professional body conferences.

### Data collection

The questionnaire was anonymous, self-administered and of a cross-sectional observational design. A mixture of open-ended, closed-ended dichotomous, contingency, nominal and ordinal polytomous questions were used to reduce the risk of missing data [22, 23]. The survey was open from January 1st to October 31st 2016.

### Data analysis

Most questions within the survey required selection of 1 of a series of fixed answers but also allowed a choice of “other” in case the fixed answers were not appropriate. If “other” was selected the responder was then able to provide a free text answer. Responses under this option were processed by seeking to reallocate them within the fixed answers (e.g. where the same terms as a fixed answer were used), and where this was not appropriate, group them into new categories. To this end, Quirkos, a qualitative data analysis software tool (Quirkos Limited, Scotland), was used to ensure objective and systematic grouping of responses by specific key words.

There were six questions where the answer was open narrative and Quirkos was used in the same way to identify groups of responses.

Descriptive statistics were derived (e.g. frequency, percentage, range). Chi squared tests were performed to compare responses from the three professional disciplines.

## Results

### Responders

A total of 512 responses were received and 13 were removed (1 patient, 1 student, 1 occupational therapist and 10 from outside the United Kingdom). The data (499 responses) comprised responses from 357 podiatrists, 93 orthotists and 49 physiotherapists.

**Fig. 1:**
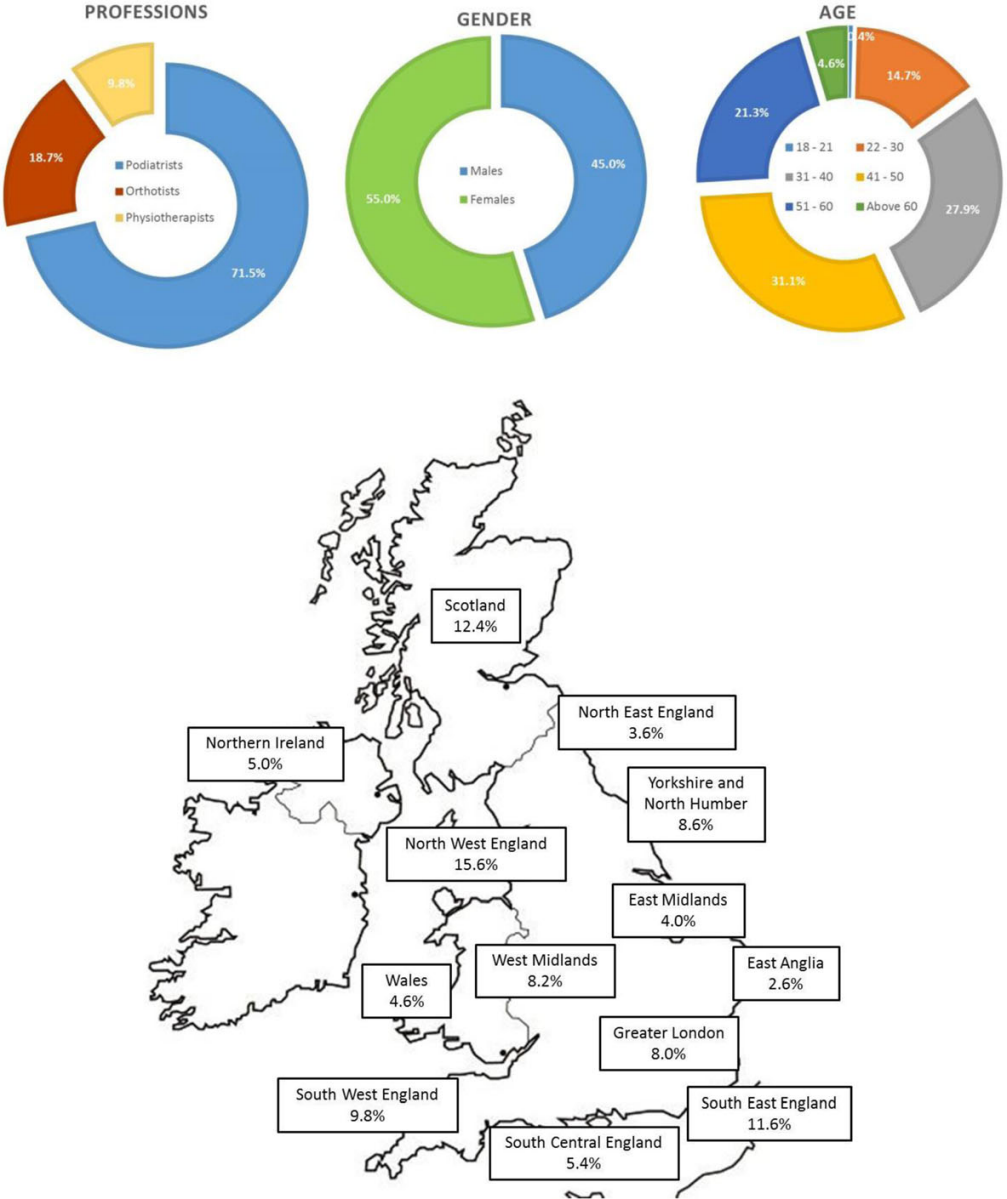
% of responders in each profession and their gender, age and location

The most frequent age range was 41 to 50 years old (31.1%) followed by 31–40 (27.9%) (Fig. 1), and 225 of responders were male (45.1%). There were 387 responses from England, 62 from Scotland, 25 from Northern Ireland and 23 from Wales (two responders did not indicate their location). The top three areas for responses were North West (15.6%) and South East England (11.6%), and Scotland (12.4%), with all other areas accounting for <10% of responders.

In total 18% of responders qualified after 2010, with 10.2–15.6% qualifying in each 5-year epoch between 1981 and 2009 (5.0% qualified pre 1980). A degree qualification was the most common highest qualification (56.5% of responders), with post graduate diploma/certificate for 27.9, and 2.6% had doctorates. A higher diploma was the highest qualification for 11% (i.e. sub degree level).

### Working context

The primary work context (i.e. >50.0% of time spent in the provision of foot orthoses) was solely the National Health Service for 52.9% of responders, was in a ‘self employed’/ private practice capacity for 23.4%, and was employed within a company providing National Health Service services for 7.8%. A further 4% worked for a private company and 1.6% for a university. A total of 10% of responders reported mixed practice between National Health Service and private practice (with more time spend in the former in most cases).

The type of service, patient groups treated with orthoses, patient groups receiving prescription footwear and additional training undertaken since qualifying are detailed in Table 1. Responders were mainly working in podiatry, musculoskeletal and orthotic departments, and treating musculoskeletal, arthritis and sports related issues, plus issues related to diabetes and neurology. The most common training topics were lower limb biomechanics (80.0% of responders), gait analysis (60.1%) and orthosis prescription (57.0%).

**Table 1:** % of responders indicating type of service in which they work, patient groups treated, patient groups receiving prescription footwear and training undertaken since qualifying

Type of service	Patient group	Patient group receiving prescription footwear	Additional training undertaking since qualifying
61.1% podiatry	90.4% MSK	21.4% diabetes	80% lower limb biomechanics
31.1% MSK	72.1% OA	17.6% arthritis	60.1% gait analysis
23.8% orthotics	63.7% sport	17.4% other high-risk	57.5% orthoses prescription
10.2% physiotherapy	63.3% diabetes	15.8% OA	42.3% sports injuries
8.2% surgery	59.1% arthritis	14.6% MSK	31.5% podopaediatrics
5.2% rheumatology	50.5% paediatrics	12.4% neuro adult	30.7% orthopaedics
3.2% CATS	45.5% adult neurology	10.2% neuro paediatrics	29.7% specialist footwear
2.4% diabetes	39.7% other high risk (e.g. stroke patients)	9.0% general paediatrics	26.7% steroid injection therapy
2.4% other	33.3% falls prevention	8.4% falls	26.3% manipulation
	3.6% “other” including post-surgery		21.0% neurology
			18.0% strength and conditioning training
			7.6% no training
			3.4% alternative therapies
			2.8% high risk population
			2.4% surgery
			1.2% diagnosis techniques
			2.2% other training

*CATS* Clinical Assessment and Treatment Service, *MSK* Musculoskeletal, *OA* osteoarthritis

A total of 27.1% of responders were able to prescribe footwear as well as foot orthoses. Diabetes, arthritis and other high risk patients were the top 3 groups receiving footwear. Of those who did not provide prescription footwear, 46.3% had access to a footwear service (25.1% did not respond to this question).

The percentage of the week spent prescribing orthoses, duration of the assessment, number of orthoses prescribed per month, and number of patients with prior insoles, are detailed in Table 2. Of the responders, 39.5% spent less than 10.0% of their week providing orthoses and 80.6% of responders provide ≤50 pairs of orthoses a month. Most responders had at least 30 min for an assessment appointment.

Of the responders 45.5% received referrals that asked them to assess the patient and decide if foot orthoses were appropriate, and 37.5% received referrals asking for an assessment of the lower limb or a specific condition, but did not refer to treatment options. Some 13.4% received referrals that advised the prescription of a foot orthosis.

The treatment objectives and outcomes expected by patients are detailed in Table 2. Relief of pain was the most common clinician objective of treatment, followed by functional control and pressure relief. Patients expected changes in pain, levels of activity / types of physical activity, and prevention of injury.

**Table 2:** Percent of week spent providing orthoses, time available for assessing patients, the number of orthoses provided per month, provision of second pair of orthoses, patients with prior use of orthoses, and clinician and patient objectives. All data are % all responders

% of week spent providing orthoses	Duration of assessment appointment	Number of orthoses per month and second pairs	Patient with prior orthoses	Clinician objectives	Patient objectives
39.5% <10% of week	35.9% = 30–45 mins	44.5% = 11–50 pairs	55.5% = 0–25% of patients	85.8% pain relief	68.3% pain reduction
37.5% = 11–50%	31.3% = 15–30 mins	36.1% = 1–10 pairs	29.3% =26–50%	66.9% functional control	63.1% return to sport level
20.6% = 51–90%	22.8% = 45–60 mins	14.0% = 51–100 pairs	8.0% = 51–75%	46.1% pressure relief	48.9% pain remission
2.4% = 100%	4.4% > 60 mins	5.4% = 100+ pairs	1.6% = 76–100%	22.8% rehabilitation (>6 months)	37.3% return to exercise
	4.2% < 15 mins	*44.1% = no 2nd pair < 3 months of 1st pair*	5.6% = did not know	21.2% accommodation of deformity	31.1% injury prevention
		*40.3% = 10*–*30% received 2nd pair*		19.4% ulcer prevention	9.2% return to work
		*<9.0% = 30*–*70% received 2nd pair*		16.2% rehabilitation (<6 months)	6.4% return to preferred footwear
		*<5.0% = 71*–*99% received 2nd pair*		14.6% stability	4.8% fall prevention
		*1.8% = 2nd pair to all patients*			18.0% did not know

**Table 3:** % of responders using each material for prefabricated orthoses, modifying prefabricated orthoses, the number of prefabricated orthoses from which responders choose, and satisfaction with choices of prefabricated orthoses

Prefabricated orthoses			
Material	Modified	Range	Choice
77.8% medium density EVA	34.4% modified 51–80%	35.7% choice of 2–5 orthoses	77.4% satisfied with range
52.1% rigid plastics	27.5% modified 11–50%	32.5% choice of > 10	13.8% not satisfied
46.9% high density EVA	10.8% modified 81–90%	17.6% choice of 5–10.	8.8% did not answer
38.3% low density EVA	10.4% modified 1–10%	2.4% had 1 design to use	68.7% of the responders had influence over the range available.
11.2% carbon fibre	5.8% modified all		
3.2% cushioning’ orthoses	3.2% never modified		
	6.8% did not answer		

*EVA* ethylene vinyl acetate

**Table 4:** % of responders using each material for customised orthoses, modifying customised orthoses, using each method of capturing foot shape and using each method of manufacture

Customised orthoses			
Material	Modified	Shape capture	Manufacture method
62.3% medium density EVA	40.9% modified ≤20%	54.1% foam boxes	29.1% handmade
51.7% rigid plastics	19.4% modified 21–99%	39.9% plaster cast	28.7% CAD/CAM
43.1% high density EVA	2.6% modified all	14.2% scanner	5.4% did not know
28.1% low density EVA	11.4% never modified (25.7% did not answer).	9.8% direct measures	4.6% mix of both approaches
25.5% carbon fibre		3.0% did not take foot impressions.	27.1% did not answer.
(<3.0%, used polyurethane, cushioning, and 3D printed orthoses)			

*CAD/CAM* computer aided design/computer aided manufacture, *EVA* ethylene vinyl acetate

In terms of facilities available to support services, 81.2% of responders had use of a corridor, 57.7% tools to modify orthoses, 30–33.0% one of the following: ovens, gym, imaging (such as X-Rays or ultrasound), vacuum formers, fume cupboards, plantar pressure measurement devices and treadmills. Some 23.2% of responders had access to video analysis and 18.2% access to CAD/CAM.

#### Orthoses provided

When asked about customised orthoses, 16.0% of the responders stated they did not provide customised orthoses at all and 5.0% provided only customised orthoses. Of the responders 37.9% indicated that up to 30% of the orthoses they provided were customised, 20.2% indicated use of customised for up to 31–60% of patients, and 20.8% provided customised orthoses for 61–99% of patients.

When asked about prefabricated orthoses, 5.8% of responders said they never provided prefabricated orthoses, while 10.0% provided only prefabricated orthoses. Of the orthoses that were provided, 43.7% of responders stated that up to 30% were prefabricated, 13.8% stated 31–60% were prefabricated, and 26.7% stated 61–99% were prefabricated. .

Cost (23.6% of responders) and time (21.5%) were the primary reasons for choosing prefabricated over a customised orthosis. Performance (12.8%), availability and ease to get another pair exactly the same (8.9%), and fit with footwear (5.5%), were other common reasons.

The ability to accurately adapt the orthosis design (14.8%), an ability to accommodate deformity (13.4%) and suitability for high-risk patients (12.4%) were the top 3 reasons for choosing customised over prefabricated orthoses. Better control (11.4%), an ability to select a range of materials (9.0%) and durability (7.9%) were also important issues.

The main comments with respect to orthotic devices and services were untrained practitioners (15.2%), budget and time (each 12.2%), and shoe fitting (9.1%). Other issues included communication with external providers (5.7%), patient expectation and compliance (5.6%), and poor evidence base (3.2%).

Recommending retail orthoses was reported by 53.3% of responders, 33.7% indicating this was for plantar fasciitis (16.6% separately for heel pain), 21.6% for over pronation, 13.6% for osteoarthritis foot pain, 11.2% for Achilles pain, 10.4% for a Morton’s neuroma, and 8% for knee arthritis (7.8% other conditions combined such as tendinosis, synovitis, bunions).

### Prefabricated orthoses

Prefabricated foot orthoses were used by 93% of responders. The reasons for not using prefabricated foot orthoses included, not being specific enough for the feet of each patient (47.2%), budget (27.8%) and service policies (22.2%) (2.8% listed other reasons).

The material used for prefabricated orthoses, % of orthoses modified, number of orthoses from which to choose, and satisfaction with choices available are detailed in Table 3. Ethylene vinyl acetate and rigid plastic were most common materials, >88% of responders modified orthoses, 50.0% of responders had five or more designs to choose from, and most responders were happy with the range available to them and had influence over it.

A second pair of prefabricated orthoses was provided whenever required by 61.9% of responders, with a further 18.4% stating once a year and 6.4% offered no replacement of prefabricated orthoses (5.0% replaced orthoses every 2 years).

Discussion with colleagues was the most common factor influencing choice of prefabricated orthoses (61.1%), followed by patient satisfaction (23.2%) and audit (20.8%).

### Customised orthoses

A total of 74.9% of responders provided customised foot orthoses as part of their practice. The materials used, % of orthoses modified, method of capturing foot shape and method of manufacture are detailed in Table 4.

Ethylene vinyl acetate and rigid plastic were the most common materials for a customised orthosis shell, with the most popular top covers being Poron (60.9% of responders), low density Ethylene vinyl acetate (44.1%), leather (37.9%), fabric (26.5%), suede (10.4%) and plastozote (9.0%). More than 60% of responders modified some customised orthoses (only 11.6% never modified customised orthoses), and 48.9% had access to an orthotic laboratory or tools to make orthotic modifications (24.0% did not, 2.0% found the question non-applicable to them and 25.1% did not answer). Foam impression boxes and plaster casts were the most common methods to capture foot shape.

Manual manufacture and computer aided design/manufacture were used in almost equal measure. Manufacture took place in a commercial company (43.1% of responders), or within the responders organisation (22.6%), or in the company the responders worked for (5.2%). For those using commercial providers delivery was more than 10 days for 46.6% of responders (23.6% >14 days), less than 10 days for 13.6% (9.8% did not use commercial companies), and 29.1% did not respond.

There were restrictions on providing customised orthoses for 17.4% of responders (26.7% did not answer the question). A second pair was provided when needed by 55.5% of responders, once a year (8.0%) or every 2 years (4.0%) and 3.8% did not replace customised orthoses.

### Other aspects of practice

Interventions other than foot orthoses were provided by 97.0% of responders. Footwear advice was provided by 89.9% and footwear by 33.7%. Exercise plans were provided by 87.5%, taping by 56.3%, mobilisation by 32%, manipulation by 25.0%, and steroid injections by 25.1%. Acupuncture, trigger point pressure, and ultrasound were also provided (all less than 15.0%).

Advice on orthoses use was provided by 97.0% of responders (3.0% said not applicable), and this was both written and verbal information for 73.3% (only verbal 27.1%, only written 5.5%). Use of online and video information was reported by <1% of responders.

Of the responders 62.7% never sent an orthosis direct to a patient, 27.6% sent 10–30% of their orthoses direct to patients (i.e. no fitting) and 3% sent more than 60% of orthoses direct to patients. Patient progress with orthoses was reviewed by 76% of responders (22.0% did not) and of these, 72.2% involved a further clinical appointment, 15.1% a telephone review, and 1.4% online communication.

Outcomes were measured or monitored by 38.0% of responders. This was most commonly a pain scale (23.5%) (eg. Visual Analogue Scale), assessment of foot biomechanics (6.1%) (e.g. video gait analysis, plantar pressure analysis, assessment of joint range of motion), patient feedback questionnaires (4.9%), audits (1.0%) and 3.0% reported other methods (e.g. new ulcer).

Use of different orthoses was most often driven by information found via catalogues (57.6%), colleagues (57.6%), online information (55.5%), conferences (51.6%) and journal papers (49.0%) (10.2% reported this was not applicable to them).

### Changes in practice

The two main factors influencing practice were conferences and research publications (15.2% of responders), followed by further training (14.9%). Discussion with colleagues also influenced changes in practice (11.2%). Budget restraints (10.1%), patient feedback and requests (10.1%) and experience (8.4%) also had an influence. An increase in the quality and variety of prefabricated orthoses had led to their greater use (6.3%), as well as changes in the availability of devices and materials from commercial companies (5.4%). Other factors such as new technologies, local policies or manufacture time had influence on changes in clinical practice (each mentioned by <5.0% of responders).

Over the next 5 years the main factor that would change practice was budget (15.7% of responders) with an expectation of limits on treatments they could offer. Responders also thought that new technologies will be available (12.5%) and be incorporated into clinical practice (e.g. 3D printing, computer aided design and milling). A change in the category of patient presenting to practitioners was also raised as an issue, with orthotic practice focusing more on specialities, such as ‘high risk’ patients, musculoskeletal or sports (8.2%). Of the responders 8.6% expected more referrals and lower waiting times for appointments, compared to 2.1% who predicted the opposite. Other future expectations were more choice of devices and/or materials (4.4%), better education and more realistic expectations from patients (each mentioned by 4.2%), enhanced audits and outcome measures (3.5%), more research to evidence treatments (3.4%) and an increase use of other therapies to reduce costs (3.3%). Some thought they would prescribe more prefabricated orthoses (3.4%) compared to 2.1% who expected to prescribe more customised orthoses.

### Other comments

Of the 499 responders 116 took the opportunity to offer further ‘free text’ comments relevant to orthotic practice and the survey (i.e. 23.2% of the sample). Of these 116, 30 stated they had no further additions to make, 15 provided comments that were not relevant and a further 15 provided responses that could not be grouped with any other responses.

The most frequent comment from the remaining 56 responders (11.0% of all responders to the survey) was a concern over the high number of untrained practitioners prescribing orthoses (mentioned by 8/56), followed by a lack of standardised protocols (7/56) and the need for more multidisciplinary teams (5/56). Other comments included the need for more research, better training, being open minded towards other therapies, reducing the cost of orthoses, and easier access to new orthoses (mentioned by <5/56 in each case).

### Statistical outcomes

There were statistically significant differences between the three professions (*p* < 0.037) for all 49 quantitative questions. However, not all responses within each question were different between professions. For example, there were statistically significant differences in work “department” between three professions, but not for every different department option (e.g. no differences between professions for departments of diabetes, occupational therapy and rheumatology, but there were differences for departments of podiatry, physiotherapy and surgical appliances and orthotics).

## Discussion

The purpose of this survey was to provide data useful to various stakeholders and support policy initiatives [24]. For example, practitioners could be empowered to reflect on their practice within their specific working context. Managers could use the data to contextualise the practice of their staff and service model. Researchers could focus on issues that are relevant to real world practice. Industry could better understand factors affecting orthotic choice and practices. Finally, national policy makers could be able to see, for the first time, the scope of foot orthotic use and the role of practitioners in contributing to improved health.

The age profile of our responders reflects the wider National Health Service workforce very well [25]. Recent scoping of the orthotist workforce [26] suggests approximately 350 full time equivalents in active practice and our 93 respondents represents a good proportion of this profession. There are far more physiotherapists and podiatrists (52,500 and 13,000 respectively [27]), but only some physiotherapists work on the foot, and perhaps only some of these use orthoses. The physiotherapy special interest group Association of Foot and Ankle Physiotherapists and other Allied Health Professionals had approximately 500 members at the time of sampling [28]. For podiatry, whilst the foot is the specific scope of practice, orthoses may not be used as a treatment strategy by all.

### Working context

Provision of foot orthoses is distributed across public and private sectors. That only 52.9% worked mainly in the National Health Service points to significant private sector provision, especially since some of these will also work in the private sector to some degree. This may explain difficulties in getting quality data on foot orthosis provision. Even within a public sector context, multiple professions and departments are involved, and patient groups too. Health organisations could therefore find it difficult to identify the scale and nature of its provision of foot orthoses. Centralised support is required to ensure cross sector, discipline and department data capture. Some data may already exist in organisations that pay for services (e.g. clinical commissioning groups) or in procurement data. However, this data would be very limited (e.g. foot orthosis used yes or no) rather than profiling the use as we have here.

Provision occurs in both uni and multidisciplinary environments, perhaps reflecting the wide range of clinical need in the patients concerned, from individual musculoskeletal complaints (e.g heel pain), to multi-faceted issues such as falls. Also, foot orthoses are not the only intervention offered by the responders. This could be useful since the same practitioner can provide multiple interventions without patients needing to have appointments with other practitioners. Common practices between health professions is in line with current national policy to remove restrictive boundaries between professions [24].

Most responders (80.0%) spend <50% of their time providing foot orthoses and approximately 40% of providers spend <10% of their time providing foot orthoses. This concurs with 36.1% of responders prescribing less than 10 pairs of foot orthoses per month. It would appear that practice is spread thin across a large number of practitioners, which is likely to be an efficient use of staff and provides quicker access to care.

Footwear advice is almost ubiquitous and demonstrates recognition of the importance of footwear in foot orthotic practice and the need to educate patients as part of a wider strategy to prevent and treat foot problems [29]. Orthotic effect and clinical outcomes are sensitive to footwear choice [30] and thus where foot orthoses are provided, an ability to manage footwear provision might also be expected. That only 27.1% of responders prescribed footwear may be at odds with this. However, equally, it might be that clinical need does not warrant individualised footwear in many cases. Whilst there is good evidence of other complementary interventions being used (e.g. taping, manipulation), footwear prescription was comparatively less common, comparable to provision of steroid injections for example. This seems a potential anomaly given the fundamental importance of footwear in influencing orthotic effect and the comparatively occasional need for injections.

### Patients receiving foot orthoses

The patients receiving foot orthoses reflect national health priorities. There is treatment of specific critical clinical episodes within several chronic diseases (e.g foot ulcers in diabetes), contribution to rehabilitation (e.g. return to normal physical activities including work), as well as management of short term episodes of clinical foot pain (e.g. plantar heel pain). Common use of orthoses in sports applications (>60% of providers) demonstrates a contribution to the active healthy living agenda too. Coupled with the focus on pain relief and the fact that a return to activities and work was an objective for patients, foot orthoses are being used in areas that appear to assist in the maintenance of the health of the nation and its workforce.

### Types of orthoses

Prefabricated orthoses are used in the main although the majority (>95%) undergo some form of modification process and are thus in fact *customised* to individual patients. Even customised orthoses (i.e. made using information on patient foot shape) are routinely modified. This apparent frequent failure of the customised orthoses design suggests the process may need to become more systematic such that errors are less common. The match between orthotic and foot shape was the main reason for using customised orthoses and suggests geometry is a key driver for orthoses design. This is reinforced by the fact that the materials used for prefabricated and customised orthoses are very similar (medium density ethylene vinyl acetate and rigid plastics) and thus the key point of difference is geometry. Foot shape (from which customised orthosis shape is derived) was captured using impression boxes and plaster casting by the majority of responders (94.0%), both of which have been shown to be error prone and less repeatable than 3D scanning [31]. This may lead to unnecessary errors in orthotic geometry that later require correction and might explain the frequent modifications to customised orthoses reported. However, whilst there is evidence that change in orthotic geometry affects foot motion and pressure in fairly systematic ways [32, 33], there is no evidence that these adjustments result in proportional changes in clinical outcomes. This questions the need for routinely adjusting orthotic shape and standardisation of practice was one of the factors raised as a future priority.

In contrast to the evidence of routine adjustment of orthoses, 53.0% of responders had recommended retail orthoses, which necessarily have a fixed shape. This also indicates that there could be significant under reported self-provision of foot orthoses. Plantar heel pain and plantar fasciitis were the most common reasons, but treatment of “over pronation” was also reported. Several authors have failed to find evidence of pronation being a problem [34, 35] or challenged the notion of over pronation [36], and indeed it is a normal foot movement [37]. This undermines the use of orthoses to modify foot pronation in the absence of clinical symptoms.

### Issues for the future

Skills and training were identified as important issues by responders. The professions concerned are currently facing changes in funding of undergraduate training and the ability of potentially small caseloads to maintain and develop foot orthotic skills warrants monitoring. Supporting skills in allied health professionals is a recognised national priority [24] and many responders already had post graduate training to help maintain or extend the capacity and capability of the foot orthoses workforce. This could be especially important given growth in clinical populations that benefit from orthoses use (e.g. diabetes, older people).

A need for improved evidence to evaluate and improve orthotic services was identified and is in line with national policy [24]. The diversity of intended outcomes (beyond improvement in pain) and relatively low use of outcome measures (used by 38.0%) perhaps indicates a need to develop orthoses specific outcome measures, or at least ensure use of outcome measures that are sensitive/specific to intended orthotic effects. The need for appropriate outcome data would benefit from national support and there are already precedents for the professions concerned (e.g. the Podiatric and Surgical Clinical Outcome Measurement, which covers more than 12,500 treatments across 100 centres in 2016 alone). Using outcome measures is an explicit requirement of professional standards and current national policy [38].

Differences between the three professions completing the survey were identified in almost all aspects of practice. This may relate to issues concerning training, scope or nature of practice and work place, the different contractual arrangement for the professions, and the scaling of specialism towards the foot (podiatrists) and orthoses (orthotists), versus the whole body (physiotherapists and orthotists). This resonates with recent policy priorities to support integration of areas of practice in ways that are patient centric rather than beholden to historical service or professional boundaries [24]. Differences between professions are covered in separate articles to allow sufficient scope for reporting and discussion of data.

#### Limitations

There are several limitations to our work. We took a pragmatic approach to sampling and generated responses from the three professions we assumed were related to foot orthosis provision. However, we are unable to quantify what percentage of the actual practitioner population we reached and professions such as occupational therapy could also be involved.

There were several places in the survey where we had relatively high numbers of nil responses, or parts with discrepancies between results when the same topic was questioned through different perspectives (e.g. 16.0% of responders stated they did not provide customised orthoses when asked about customised orthoses, yet only 10.0% said they provided only prefabricated orthoses i.e. no customised orthoses, when asked about use of prefabricated orthoses). It could be that some responders did not think the questions leant themselves to their practice and chose to ignore some questions. However, relatively few responders used the free text section at the end to express further comments, which might have been a good place for anyone who thought their practice was not well represented to share their views. We therefore think the questionnaire was comprehensive in its coverage of issues. However, it might equally be that in being thorough some responders were fatigued and selective in the level of detail they provided. There is inevitably a balance between the desire for more data on the issue being investigated and the volume of questions that can be asked, without risking participants loosing focus. Our pilot work allowed us to work directly with people in practice and prioritise content appropriately. It could be argued that the pilot work did not provide equal representation from physiotherapists in particular, although there was no pattern in the responses to the final survey to suggest that the practice of any one profession was less well represented than that of another.

## Conclusion

Foot orthoses in the United Kingdom are provided by podiatrists, orthotists and physiotherapists to a wide range of clinical populations, including those of high priority on the health policy agenda. However, provision is spread across health care organisations making collection and use of data for innovation of services challenging. There is, however, recognition for the need for evidence to support change. A wide range of orthotic devices and practices are employed and different professions provide foot orthoses in different ways. This perhaps points to persistence of professional boundaries. If so this would be at odds with national policy that promotes integration of care across traditional boundaries. The differences between professions are the topic of the second article in this series.

## Additional file

Additional file 1: Appendix A. Survey of foot orthoses provision in the UK. (DOCX 28 kb)
