# Supplementary material for: National profile of foot orthotic provision in the United Kingdom, part 1: practitioners and scope of practice
Source: J Foot Ankle Res. 2017 Aug 1;10:35. doi: 10.1186/s13047-017-0215-4 (PMC5540424; doi:10.1186/s13047-017-0215-4)
Supplement: Additional file 1: Appendix A. — Survey of foot orthoses provision in the UK. (DOCX 28 kb) [file 13047_2017_215_MOESM1_ESM.docx]

# **Survey of foot orthoses provision in the UK.**

# **Part 1 -About you**

1. Are you:
   1. Male
   2. Female
2. What is your age? Please select one option:
   1. 18-21 years
   2. 22-30 years
   3. 31-40 years
   4. 41-50 years
   5. 51-60 years
   6. Above 60 years
3. What YEAR did you qualify? Please select the year that you obtained your professional qualification. Thank you.
   1. 1968
   2. 1969
   3. 1970
   4. 1971
   5. 1972
   6. 1973
   7. 1974
   8. 1975
   9. 1976
   10. 1977
   11. 1978
   12. 1979
   13. 1980
   14. 1981
   15. 1982
   16. 1983
   17. 1984
   18. 1985
   19. 1986
   20. 1987
   21. 1988
   22. 1989
   23. 1990
   24. 1991
   25. 1992
   26. 1993
   27. 1994
   28. 1995
   29. 1996
   30. 1997
   31. 1998
   32. 1999
   33. 2000
   34. 2001
   35. 2002
   36. 2003
   37. 2004
   38. 2005
   39. 2006
   40. 2007
   41. 2008
   42. 2009
   43. 2010
   44. 2011
   45. 2012
   46. 2013
   47. 2014
   48. 2015
   49. Other, please specify:
4. What is your HIGHEST level of qualification?
   1. Higher National Diploma
   2. Bachelors Degree
   3. Masters Degree
   4. Doctorate (PhD)
   5. Other, please specify:
5. Are you a registered: (please select the response appropriate to you)
   1. Podiatrist
   2. Orthotist
   3. Physiotherapist
   4. Occupational Therapist
   5. Other, please specify:
6. Since qualifying in your profession have you undertaken additional training related to the lower limb in any of the following areas. Please select all that apply to you.
   1. Lower Limb Biomechanics
   2. Gait analysis
   3. Orthopaedics
   4. Specialist footwear
   5. Podopaediatrics
   6. Sports Injuries
   7. Strength and Conditioning
   8. Training
   9. Neurology
   10. Foot Orthoses Prescription
   11. Manipulation
   12. Steroid Injection Therapy
   13. I have not undertaken any additional training
   14. Other, please specify:
7. Which geographic location do you work within? Select one answer that best represents where you work for the majority of your working week.
   1. South East England
   2. North West England
   3. South West England
   4. Greater London
   5. West Midlands
   6. East Anglia
   7. Yorkshire and North Humber
   8. East Midlands
   9. South Central England
   10. North East England
   11. Scotland
   12. Northern Ireland
   13. Wales
   14. Republic of Ireland

# **Part 2: Your Practice**

1. Who do you spend the MAJORITY of your time working for in the provision of foot orthoses? Select only ONE answer that best describes you.
   1. The NHS
   2. As a self-employed Independent
   3. (Private) practitioner
   4. A private or commercial company
   5. A private or commercial company providing NHS services
   6. 50-50 split in NHS and Private Practice
   7. 60-40 split in NHS and Private Practice
   8. 70-30 split in NHS and Private Practice
   9. 60-40 split in Private Practice and NHS
   10. 70-30 split in Private Practice and NHS
   11. Other, please specify:
2. What department do you work in when providing foot orthoses? Select all that apply.
   1. MSK
   2. CATS
   3. Podiatry
   4. Physiotherapy
   5. Occupational Therapy
   6. Rheumatology
   7. Surgical Appliances
   8. Orthotics
   9. Other, please specify:
3. What facilities are available to you that support your use of foot orthoses? Select all that apply.
   1. Grinding Machines
   2. Vacuum Former
   3. Ovens
   4. Fume Cupboard
   5. Treadmill
   6. Pressure Plates
   7. 2D/3D - Video analysis
   8. CAD/CAM
   9. Imaging Facilities (diagnostic utrasound-access to plain film x-ray/MRI)
   10. Gym facilities suitable for assessment/observing gait
   11. Corridor for observing gait
   12. Other, please specify:
4. What percentage of your working week is spent providing foot orthoses? Please select the percentage that best reflects you. As a guide each 10% increment represents a half day.
   1. Less than 10%
   2. 10%
   3. 20%
   4. 30%
   5. 40%
   6. 50%
   7. 60%
   8. 70%
   9. 80%
   10. 90%
   11. 100%

# **Part 3: Your Patients**

1. What percentage of your patients have prior experience of (any) foot orthoses before you treated them? Please select the percentage range that is most reflective of your practice.
   1. 0-5%
   2. 6-10%
   3. 11-15%
   4. 16-20%
   5. 21-25%
   6. 26-30%
   7. 31-35%
   8. 36-40%
   9. 41-45%
   10. 46-50%
   11. 51-55%
   12. 56-60%
   13. 61-65%
   14. 66-70%
   15. 71-75%
   16. 76-80%
   17. 81-85%
   18. 86-90%
   19. 91-95%
   20. 96-100%
   21. I don't know
2. Which patient groups do you treat using foot orthoses? Select all that apply. If you feel there is an option that is not represented please use the "other" response to give your answer.
   1. General Musculoskeletal
   2. Diabetes
   3. Inflammatory Arthritis
   4. Osteoarthritis
   5. General Paediatric
   6. Neurological (adult)
   7. Neurological (paediatric)
   8. Sports Injuries
   9. Other High Risk (e.g
   10. Peripheral Arterial Disease
   11. patients)
   12. Falls-related Patients
   13. Other, please specify:
3. What are YOUR THREE MAIN treatment objectives for your patient groups? Select three items from the following. If you feel there is another option that you would choose that does not appear here, please add it into the 'other' section.
   1. Pain relief
   2. Pressure relief
   3. Functional control
   4. Accommodate Deformity
   5. Proprioception/stability control
   6. Ulcer prevention
   7. Short term rehabilitation (less than 6 months)
   8. Long term rehabilitation (more than 6 months)
   9. Other, please specify:
4. What OUTCOMES do PATIENTS tell you they want to achieve? Please select the THREE main outcomes from the options below. If there is an option that we have not considered please select 'other' to enter the alternative outcome.
   1. Pain reduction
   2. To be pain free
   3. Return to sporting activity
   4. Return to a certain level of activity (non-sports related)
   5. Return to work
   6. Prevent injury (e.g ulceration or musculoskeletal)
   7. Return to a certain type of footwear
   8. Prevent falls
   9. Patient not sure what they want from Foot Orthoses
   10. Other, please specify:

# **Part 4: Your Practice**

**Sub section 4.1: General Prescribing habits**

1. How many pairs of foot orthoses do you provide (both bespoke/custom and prefabricated) per month in all your working contexts (i.e in the NHS, commercial sector and private practice)?
   1. 1-10
   2. 11-50
   3. 51-100
   4. 100+
2. What percentage of patients would receive a second pair of foot orthoses at or around the same time (within 3 months) as the 1st pair? Please select one response.
   1. No patients receive 2 pairs
   2. 10-20%
   3. 21-30%
   4. 31-40%
   5. 41-50%
   6. 51-60%
   7. 61-70%
   8. 71-80%
   9. 81-90%
   10. 91-99%
   11. All patients receive 2 pairs
3. What do MOST of the referrals request? Please select one response that best reflects you.
   1. Assessment of patients lower limb and decide if FO is appropriate
   2. Requests you to prescribe an FO
   3. Assessment of lower limb /condition without reference to a treatment choice e.g due to back pain
   4. Other, please specify:
4. Do you prescribe and fit footwear as well as foot orthoses?
   1. Yes
   2. No

19.a. If you answered 'yes' which patient groups do YOU provide and fit footwear for? Select all that apply.

- 1. General Musculoskeletal
  2. Diabetes
  3. Inflammatory Arthritis
  4. Osteoarthritis
  5. General Paediatric
  6. Neurological (adult)
  7. Neurological (paediatric)
  8. Sports Injuries
  9. Other High Risk (e.g Peripheral Arterial Disease patients)
  10. Falls-related Patients
  11. Other, please specify:

1. If you answered 'no' to the question "Do you provide and fit prescription footwear", do you have access to a prescription footwear service if you need it?
   1. Yes
   2. No
2. Do you ever suggest specific retail orthoses that the patients might purchase themselves?
   1. Yes
   2. No

21.a If you answered 'yes', which foot conditions is this MOST COMMONLY in relation to? Please select all that apply.

- 1. Plantar Fasciitis
  2. Non-specific Heel pain
  3. Achilles tendinopathy
  4. OA related knee pain
  5. OA related foot pain
  6. Morton’s neuroma
  7. Over pronation due to excess foot joint mobility
  8. Other, please specify:

1. How long is an assessment appointment during which you might decide to provide foot orthoses? Please select one option from the choices below. If your answer is different from those provided, please use the 'other' answer response to provide your time.
   1. 0-15 minutes
   2. 15- 30 minutes
   3. 30-45 minutes
   4. 45-60 minutes
   5. 60 + minutes
   6. Other
   7. If you, please specify:
2. What percentage of the foot orthoses you provide are bespoke/custom made? Select one percentage range.
   1. 0%
   2. 5-10%
   3. 11-20%
   4. 21-30%
   5. 31-40%
   6. 41-50%
   7. 51-60%
   8. 61-70%
   9. 71-80%
   10. 81-90%
   11. 91-99%
   12. All are bespoke/custom foot orthoses
3. What percentage of the foot orthoses that you provide are prefabricated? Please select one percentage range.
   1. 0%
   2. 5-10%
   3. 11-20%
   4. 21-30%
   5. 31-40%
   6. 41-50%
   7. 51-60%
   8. 61-70%
   9. 71-80%
   10. 81-90%
   11. 91-99%
   12. All are prefabricated foot orthoses
4. What are your top THREE reasons for using prefabricated over bespoke/custom made orthoses? Please use the text box to list 3 brief reasons.
5. What are your top THREE reasons for using bespoke/custom made orthoses over prefabricated? Please use the text box to list 3 brief reasons.
6. In the free text box below please outline (briefly) the problem with foot orthotic DEVICES or SERVICES that most need addressing?
7. Do you use prefabricated foot orthoses?
   1. Yes - proceed to question 30
   2. No - proceed to question 29.
8. If you do not use pre-fabricated foot orthoses what are the reasons for this? Please select all that apply from the responses below and use the 'other' response option to provide answers that are not given here. Then proceed to Page 8 - question 39.
   1. They are not supplied within the service I work for
   2. Due to budget restraints
   3. Other, please specify

**Sub-section 4.2: Prefabricated orthoses**

1. Of the prefabricated orthoses that you use, identify the THREE materials that form the orthotic shell in most cases. Please select only THREE options from the choices below. If we have not provided an option that you would like to select please use the 'other' answer response to give your choice(s).
   1. Low density EVA
   2. Medium Density EVA
   3. High Density EVA
   4. Polypropylene
   5. Subortholen (Polyethylene)
   6. Carbon Fibre
   7. Other, please specify:
2. In what percentage of patients do the prefabricated orthoses need some chairside/in clinic modification, such as the addition of rearfoot wedges, creation of apertures or minor alterations to the fit, prior to first fitting? Please select one response or use the 'other' response option if you have additonal information.
   1. No patients require modifications to prefab orthoses
   2. 1-10%
   3. 11-20%
   4. 21-30%
   5. 31-40%
   6. 41-50%
   7. 51-60%
   8. 61-70%
   9. 71-80%
   10. 81-90%
   11. 91-99%
   12. All patients require modifications to prefab orthoses
   13. Other, please specify:
3. Do you have access to an on-site Orthotics Laboratory or equipment such as a grinding machine to make modifications to pre-fabricated devices if required?
   1. Yes
   2. No
   3. Not applicable to me
4. What is it about one prefabricated orthotic that convinces you it is a better choice (this could be that you think it is a better fit or more effective, for example) than another choice of prefabricated orthotic? Please give a BRIEF outline in the text box below.
5. What are the typical costs in pounds sterling of prefabricated orthoses when supplied direct to YOU (the practitioner) or NHS Trust or company you work in? Please select one cost range from those below. If there is a different answer you wish to provide please use the 'other' answer response.
   1. £0.50 - £5.00
   2. £6.00 - £10.00
   3. £11.00 - £15.00
   4. £16.00 - £20.00
   5. £21.00- £25.00
   6. £26.00-£30.00
   7. £31.00-£35.00
   8. £36.00-£40.00
   9. £41.00- £45.00
   10. £46.00-£50.00
   11. More than £50
   12. Other, please specify:
6. Are you happy with the choice of prefabricated foot orthoses that are available for you to prescribe/supply?
   1. Yes
   2. No

35.a If you answered 'no', what are the reasons for this? Please give a BRIEF outline in the text box below.

1. How many types of prefabricated foot orthoses are made available for you to choose from? Please select one response.
   1. 1
   2. 2
   3. 3
   4. 4
   5. 5
   6. 6
   7. 7
   8. 8
   9. 9
   10. 10
   11. More than 10
2. Are you able to influence the choices of prefabricated orthoses that are made available to you?
   1. Yes
   2. No
   3. Not applicable to me

37.a If you answered 'yes', by what processes do you influence these choices. Please select all that apply.

- 1. Through the results of clinical audit
  2. Through the results of patient satisfaction surveys
  3. Through discussion with colleagues

1. How often are the patients provided with a new pair of prefabricated foot orthoses? Please select the response that is most reflective of your practice.
   1. Once a year
   2. Every two years
   3. Whenever they are needed
   4. We do not replace prefabricated foot orthoses
2. Do you prescribe/fit bespoke/custom foot orthoses?
   1. Yes - proceed to question 40
   2. No - proceed to question Page 9, question 52.

**Sub-section 4.3: Bespoke/ custom made Orthoses**

1. Of the bespoke/ custom made foot orthoses that you use, identify the THREE materials that form the orthotic shell in MOST cases. Please select THREE from the responses below or use the 'other' option if there is a response you wish to give that we have not provided.
   1. High density EVA
   2. Medium density EVA
   3. Low density EVA
   4. Polypropylene
   5. Carbon Fibre
   6. Other, please specify:
2. Of the bespoke/ custom made foot orthoses that you use, identify the THREE materials that form the orthotic top cover in MOST cases. Please select THREE materials from the responses below or use the 'other' option if there is a response you wish to give that we have not provided.
   1. Low density EVA
   2. Poron
   3. PPT
   4. Fabric
   5. Leather
   6. Suede
   7. Plastazote
   8. Other, please specify:
3. In what percentage of patients do the bespoke / custom made foot orthoses need some chairside/in clinic modification prior to or at first fitting. Please select ONE response from the choices below.
   1. No patients require modifications to bespoke/custom made orthoses
   2. 1-10%
   3. 11-20%
   4. 21-30%
   5. 31-40%
   6. 41-50%
   7. 51-60%
   8. 61-70%
   9. 71-80%
   10. 81-90%
   11. 91-99%
   12. All patients require modifications to bespoke/custom made orthoses
4. Do you have access to an on-site Orthotics Laboratory or equipment such as a grinding machine to make modifications to bespoke devices if required?
   1. Yes
   2. No
   3. Not applicable to me
5. What methods do you TYPICALLY use to capture foot shape? Please select all that apply. If there are other responses that we have not considered please, select 'other' provide your response.
   1. Foam Impression Box
   2. Plaster of Paris bandage
   3. Digital Scanning technology
   4. Direct measures of foot dimensions
   5. I don’t take foot impressions
   6. Other, please specify
6. Are the bespoke/ custom made orthoses you use handmade (i.e. vacuum formed to a cast) or milled using CAD/CAM? Please select one response.
   1. Hand made (vacuum formed to a
   2. cast)
   3. Milled using CAD/CAM
   4. I don't know
   5. Not applicable to me
   6. Other, please specify
7. Where are the bespoke/custom made orthoses you prescribe made? Please select one response.
   1. Bespoke / custom made orthoses are made 'in-house'
   2. Bespoke / custom made orthoses are made via a commercial company
   3. In-house (within a the commercial company that I work for)
   4. Not applicable to me
   5. Other, please specify
8. How long does it take for bespoke / custom made orthoses to be supplied to YOU once they have been ordered via a commercial company?
   1. 0-2 working days
   2. 3-5 working days
   3. 6-9 working days
   4. 10-14 working days
   5. More than 14 working days
   6. Not applicable. We don't use commercial companies for foot orthoses.
   7. Other, please specify:
9. If the bespoke/custom made orthoses that you use are manufactured 'In-House (on NHS facilities by NHS staff): What are the AVERAGE costs in pounds sterling, to the practitioner or NHS Trust? Please select one response from below. If your bespoke/custom devices are made ONLY by a commercial company please go to question 49.
   1. Less than £25
   2. £25 -30
   3. £31- 35
   4. £36-40
   5. £41-45
   6. £45-50
   7. £51-55
   8. £56- 60
   9. £61-65
   10. £66 -70
   11. £71 -75
   12. £76-80
   13. £80 -85
   14. £86-90
   15. £91-95
   16. £96-100
   17. More than £100
   18. Other, please specify:
10. If the bespoke/custom made orthoses that you use are manufactured via a commercial company, what are the AVERAGE costs in pounds sterling of bespoke/custom made orthoses (to the practitioner or NHS trust)? Please select the nearest value.
    1. Less than £25
    2. £25 -30
    3. £31- 35
    4. £36-40
    5. £41-45
    6. £46-50
    7. £51-55
    8. £56- 60
    9. £61-65
    10. £66 -70
    11. £71 -75
    12. £76-80
    13. £80 -85
    14. £86-90
    15. £91-95
    16. £96-100
    17. More than £100
    18. Other, please specify:
11. Are there any restrictions upon your ability to provide bespoke/custom made foot orthoses?
    1. Yes
    2. No
12. How often are patients provided with a new pair of bespoke/custom made foot orthoses? Please select one response or provide an alternative response using the 'other' option.
    1. Once a year
    2. Every two years
    3. Whenever they are needed
    4. We do not replace bespoke/custom made foot orthoses
    5. Other, please specify:

# **Part 5: Other Information**

1. Do you provide other treatment interventions alongside foot orthoses?
   1. Yes
   2. No
   3. Not applicable to me

52.a If you answered 'yes', what type of interventions do you provide? Please select all that apply and add in extra answers not covered here via the 'other' option.

- 1. Exercise programme (such as stretches, strengthening/conditioning)
  2. Footwear advice
  3. Footwear
  4. Acupuncture
  5. Taping
  6. Steroid Injection
  7. Manipulation
  8. Mobilisation
  9. Trigger point therapy
  10. Therapeutic Ultrasound
  11. Other, please specify:

1. Do you provide advice on how to use the foot orthoses as part of your fitting service?
   1. Yes
   2. No
   3. Not applicable to me

53.a If you answered 'yes' you give advice, please indicate how you provide the advice, and select all that apply. Provide additional responses not offered via the 'other' option.

- 1. Verbal
  2. Written
  3. Both verbal and written
  4. Other, please specify:

1. What percentage of the foot orthoses are sent directly to the patient, rather than being fitted by you? Please select the closest percentage from the list below:
   1. 0%
   2. 1-10%
   3. 11-20%
   4. 21-30%
   5. 31-40%
   6. 41-50%
   7. 51-60%
   8. 61-70%
   9. 71-80%
   10. 81-90%
   11. 91-100%
2. Do you routinely review patients for whom you provide foot orthoses?
   1. Yes
   2. No
   3. Not applicable to me

55.a If you answered 'yes' to this question, how is this undertaken? Please select all that apply. If 'no' please go to the next question.

- 1. By clinic appointment
  2. By telephone review
  3. Other, please specify:

1. Do you measure or monitor (i.e with an outcome measure tool or questionnaire) outcomes from the foot orthoses you provide?
   1. Yes
   2. No
   3. Not applicable to me

56.a If you answered 'yes' to this question please list here the outcome measurement tools or questionnaires that you use (for example: you may use a pressure measurement system or a foot specific outcome measure).

1. If you are looking for new orthotic designs or choices, where do you go to find information? Please select all those that apply. If there are options that we have not considered then please select 'other' and then include your additional responses.
   1. Orthotic manufacturer catalogues
   2. Online resources
   3. Fellow colleagues in other
   4. Trusts at Special Interest groups
   5. Through research presented at conferences
   6. Through research published in journal articles
   7. Not applicable to my role
   8. Other, please specify:
2. What factors have influenced changes to your practice in the use, manufacture and/or prescription of foot orthoses in the last five years. Please use the text box to give a brief outline.
3. What are the top TWO ways in which your practice will change in the next FIVE years (this could be the patients you treat, patients expectations, how long patients might have to wait, fittings, choices, suppliers, costs...) Please use the text box to BRIEFLY outline your answers.
4. Do you have any other comments you wish to add in relation to the prescription, supply and fitting of foot orthoses that we have not considered in this survey? Please use the text box below
